# MicroRNA-Mediated Regulation of Vascular Endothelium: From Pro-Inflammation to Atherosclerosis

**DOI:** 10.3390/ijms26135919

**Published:** 2025-06-20

**Authors:** Vinícius Rodrigues Silva, Ashraf Azar, Edmilson Ricardo Goncalves, Thatiane Cristina de Moura Nascimento, Rogerio Leone Buchaim, Daniela Vieira Buchaim, Fernando Antonio Antunes de Oliveira, Carolina Costa Nassar, Tais Mendes de Camargo, Ricardo Farinasso Caboclo, Marcelo Rodrigues da Cunha

**Affiliations:** 1 Center for Life Sciences, Pontifical Catholic University (PUC Campinas), Campinas 13034-685, Brazil; vinicius.rodrigues@puc-campinas.edu.br (V.R.S.); erg@puc-campinas.edu.br (E.R.G.); fernando.antonio@puc-campinas.edu.br (F.A.A.d.O.); carolina.cn@puccampinas.edu.br (C.C.N.); 2 Postgraduate Program in Health Sciences, Faculty of Medicine of Jundiaí (FMJ), Jundiaí 13202-550, Brazil; 3 Chemistry Department, Vanier College, Montreal, QC H4L 3X9, Canada; azara@vaniercollege.qc.ca; 4 Department of Biochemistry and Molecular Medicine, University of Montreal, Montreal, QC H3C 3J7, Canada; 5 Department of Translational Medicine, School of Medical Sciences, University of Campinas (UNICAMP), Campinas 13087-883, Brazil; t144015@dac.unicamp.br; 6 Graduate Program in Anatomy of Domestic and Wild Animals, Faculty of Veterinary Medicine and Animal Science, University of Sao Paulo (FMVZ/USP), Sao Paulo 05508-270, Brazil; rogerio@fob.usp.br (R.L.B.); danibuchaim@alumni.usp.br (D.V.B.); 7 Department of Biological Sciences, Bauru School of Dentistry (FOB/USP), University of Sao Paulo, Bauru 17012-901, Brazil; 8 Medical School, University Center of Adamantina (FAI), Adamantina 17800-000, Brazil; 9 Department of Anatomy, Dentistry School, Faculty of the Midwest Paulista (FACOP), Piratininga 17499-010, Brazil; 10 Department of Physiotherapy, São Francisco University (USF), Bragança Paulista 12916-900, Brazil; tais.camargo@usf.edu.br (T.M.d.C.); ricardo.caboclo@usf.edu.br (R.F.C.); 11 Neurobiology Study Group, Nossa Senhora do Patrocínio University Center (CEUNSP), Itu 13300-200, Brazil

**Keywords:** microRNA, atherosclerosis, vascular endothelium, inflammation, biomarkers, atherogenesis, blood diseases

## Abstract

Endothelial inflammation and atherosclerosis remain leading drivers of cardiovascular disease, yet the post-transcriptional regulators orchestrating these events are not yet completely understood. In this review, we analyse recent preclinical and clinical studies to dissect microRNA (miRNA)-mediated control of vascular endothelial biology. We describe how miR-181b-5p and miR-223 modulate NLRP3 inflammasome activation and pyroptosis, how miR-615-5p, miR-138, and miR-133a coordinate endothelial nitric oxide synthase (eNOS) activity and nitric oxide bioavailability, and how miR-33a/b, miR-150, and miR-342-3p influence lipid efflux and foam-cell formation in atherogenesis. We also discuss miRNA signatures that correlate with endothelial dysfunction in human cohorts. By integrating mechanistic pathways with emerging biomarker data, this study underscores the relevance of miRNAs as both diagnostic and potential targets in vascular diseases.

## 1. Introduction

During the first phase of the Encyclopaedia of DNA Elements (ENCODE) project, results suggested that nearly 90% of the human genome might be transcribed to proteins [1]. This estimate was later revised and adjusted to 74.7% at the conclusion of the study [2]. Furthermore, 98.5% of the human genome does not code for proteins, and only approximately 1.5% encodes proteins via messenger RNA (mRNA). Of the transcribed fraction, approximately 66% were classified as non-transcriptional or undetermined sequences, signifying an inability to encode for a specific protein [3]. There has been sufficient growing evidence that the mammalian genome is extensively transcribed into non-coding RNA (ncRNA) through various mechanisms including alternative splicing and intronic transcription, associated with cell variability, representing essential evolutionary processes and pathways [4,5,6]. A significant number of ncRNAs have been discovered in the past ten years. Over the past decade, transcriptome analyses have identified numerous novel ncRNAs, and dedicated libraries and databases continue to expand. Nevertheless, the biological functions and implications of the majority of these ncRNAs remain to be fully characterised. Current research efforts are making significant advances to help understand the functional purposes and roles of different ncRNAs [7]. As sequence catalogues grow, so does the potential for ncRNAs to serve as therapeutic targets and clinical biomarkers.

### Non-Coding RNA Classification

ncRNAs are classified according to the number of nucleotides (nt) present in their strips, identifying differences in their biological functions and the locations of activity in the cell nuclear space. Thus, they are separated into two broad groups such as short-chain ncRNAs (<200 nts) and long-chain (>200 nts) [8,9].

MicroRNAs (miRNAs) are single-stranded ncRNAs. The approximately 22 nts-based molecule was discovered in 1993 through the study of the nematode *Caenorhabditis elegans* and was subsequently named in 2001 [10,11,12]. miRNAs showed conserved phylogenetic structural characteristics found in plant and animal cells observed in mediating the regulation of protein expression by inhibiting mRNA translation or increasing the decay rate as part of the silencing process through argonaute (Argo) proteins through miRNA-induced silencing complexes (miRISCs) [13,14]. Of the several types of miRNAs, they are generally grouped into 2 groups: 1—the miRNAs that are expressed and remain intracellular, and 2—the circulating miRNAs that are expressed and are extracellular, having functional purposes in various tissues. The miRBase data, one of the largest miRNA databases, has catalogued over 48.860 mature miRNAs identified in 271 organisms showing how extensive and relevant these molecules might be with diverse biological systems [15]. In general, studies indicate that human miRNAs can target from 30% to 60% of the transcribed genes, which suggests their participation in processes such as differentiation, and cell death programming [14], and in meaningful interactions with several disease processes [16,17,18,19,20].

The involvement and implications of miRNAs can be found in all cells, tissues, and organs. In this regard, the present study aims to discuss the latest findings related to this small untranslated RNA and its role in vascular endothelium disorders, more specifically in pro-inflammation, endothelial dysfunction, and atherogenesis (Table 1).

## 2. Biogenesis and Regulation of Gene Expression

The biogenesis of miRNA is classified into two different mechanisms: canonical and non-canonical [38]. The canonical pathway starts from the transcription of primary miRNA (pri-miRNA) containing a 5′ CAP and a 3′ poly(A) tail, through the enzyme RNA polymerase II processing from intronic, exonic or intergenic regions. Part of this hairpin-shaped chain facilitates recognition for the cleavage process by a complex microprocessor, composed of nuclear enzymes of the RNase III family, particularly the class 2 ribonuclease III, Drosha, gene-specific for double-stranded RNAs. Drosha is conjugated to the protein Di-George syndrome Critical Region gene 8 (DGCR8) acting as an anchor molecule and assisting the cleavage of 11 base pairs (bp), allowing disruption in hairpin-way [39]. The pri-miRNA becomes a precursor miRNA (pre-miRNA) with an estimated 70 to 200 nts. The pre-miRNA is exported out of the nucleus towards the cytosolic mean due to the protein complex Exportin5 (Exp5) plus RAN gene-encoded protein, Ran cofactor coupled to guanosine triphosphate (GTP) [40,41]. The subsequent hydrolysis of GTP to GDP + free phosphate + energy is carried to the cytoplasm, concomitantly releasing the Exp5 protein to return to the nucleus to complete a new cycle. In parallel, the multi-domain ribonuclease Dicer enzyme associated with the transactivation response element RNA-binding protein (TRBP) cofactor changes the pre-miRNA in a duplex miRNA structure [42]. Protein members of the Argo family 1 to 4 plus TARBP2 promote the double-strand separation giving rise to a mature miRNA, consequently leading to the disposal of the other strand through the degradation pathway [43,44].

Conversely, the non-canonical mechanism is an alternative pathway using Drosha/DGCR8 activity, which appears to be assisted by Dicer, Argo and Exportin complexes [45]. However, this pathway is led by nuclear complexes called spliceosomes that act on short intronic regions (mirtrons) carrying the pre-miRNA formation, which also binds to Exp5 towards the cytoplasm where it undergoes cleavage via the Dirce/TRBP/Argo2 complex. Similar to the canonical route, one of the strands is excluded by the degradation pathway keeping the mature miRNA [46].

Proper miRNA biogenesis via Drosha–DGCR8 and Exportin-5 is essential for maintaining endothelial inflammatory homeostasis, as disruptions in these processing steps alter NF-κB activation and pro-inflammatory cytokine expression in vascular endothelium.

### MicroRNA Genes Regulation

In the gene regulation process, miRNAs may be involved in the regulation of more than one mRNA; in the same way, mRNA can also be influenced by multiple miRNAs [44,45,46,47]. We highlight the main mechanisms of miRNA gene regulation:

(A) Inhibition of translation by, (i) competition for the 5′ CAP region through cap-binding protein (CBP-mRNA) [48]; (ii) inhibition of ribosome assembly to mRNA impairment of Argo2 and translation initiation factor 6 (eIF60) connection [49]; (iii) deadenylation followed by the blockage of translation initiation through the binding of the 5′ CAP region to the poly-A tail due with Poly (A) binding protein cytoplasmic 1 (PABPC1) and eIF4G interaction [50]; (iv) premature dissociation of ribosomes for blocking the mRNA translation through the association of miRNA/mRNA/rRNA [51]; (v) reduction of the elongation speed in protein formation [52]; and (vi) proteolysis during the elongation phase, although this mechanism is still not clearly understood [53].

(B) Destabilisation of the target RNA by, (i) cleavage of the target transcript where miRNA cleaves 5′ CAP-3′ Poly (A) degrades by exoribonucleases [54]; and (ii) deadenylation followed by removal of the 5′ CAP when GW182 interaction occurs through to protein platform CCR4/NOT and DCP21/DCP2 complexes [55].

(C) Transcriptional silencing by, (i) DNA methylation, through the methyl group (CH_3_) making unfeasible the transcription factor’s activation [56]; and (ii) chromatin modification through Argo2-ncRNA acting in the promoter region where RNA polymerase II could bind to initiate the transcription process [57].

Finally, transcription induced by interaction with TATA-box binding protein (TBT) to RNA polymerase II increases three to eight times [58] and increases translation efficiency through the synthesis of microribonucleoproteins (microRNP) [59].

MiRNAs might also exert an influence on gene silencing by degrading the mRNA strand with deadenylation from the 3′-end or removing the Cap structure from the 5′-end followed by the loss of the poly-A tail. At this stage, the mRNA is destined for degradation via cytoplasmic exonuclease. In addition, the mRNA may be cleaved by the polysomal enzyme ribonuclease-1 (PMR1) acting in a specific endonucleolytic sequence [60].

Another important mechanism includes the involvement of the Argo protein, which could reduce the eukaryotic translation initiation factor 4E (elF4E) through ribosomes to the Cap structure of mRNA inducing its degradation [61]. The Argo complex competes with elF4E for connection to the Cap-type structure and can also interfere with the closed-loop structure from RNA, which is essential for the translation process [62]. Another form of action of miRNAs in gene regulation may occur in the initial phase of post-translational modification when the complex miRNP-Ago2 coupled to elF6 prevents the association of the large subunit of the ribosome with the mRNA strand in order to avoid translation [63]. The interaction between miRNA and mRNA is highly specific, determined by the seed region, located at the 5′-end, formed by 2 to 7 nt’s from the mature miRNA sequence, which binds to the 3′ UTR (3′ untranslated region)-end of the mRNA target. This relationship determines which mRNA should interact with specific miRNA, thus promoting gene silencing or destruction [64].

## 3. miRNAs Regulation in Non-Infectious Pro-Inflammation

Pro-inflammatory signalling is related to the activation of the innate immune system responsible for controlling the adaptive responses in vascular endothelium. The endogenous non-infectious substances released from dying cells or metabolic content in the cytosolic space, adenosine triphosphate (ATP), reactive oxygen species (ROS), oxidised low-density lipoprotein (ox-LDL), cardiolipin, deoxyribonucleic acid (DNA), nicotinamide adenine dinucleotide (NAD^+^), inositol-requiring enzyme-1 alpha (RE1α), amongst others [65,66] identified as damage-associated molecular patterns (DAMPs) are responsible for triggering this mechanism.

### 3.1. Sterile Inflammation DAMPs-Induced

Damage-associated molecular patterns (DAMPs) species are recognised by intracellular or extracellular cellular receptors named pattern recognition receptors (PRRs): C-type lectin receptors (CLR family); retinoic acid-inducible gene-I-like receptors (RLR family); nucleotide-binding domain-like oligomerisation receptors (NLR family); and absent-in-melanoma-2-like receptors (ALR family) are found in the vast majority of non-immune cells [67,68]. Consistent ’DAMPs’ release may induce vasoconstriction dysregulation, endothelial dysfunction and inflammation disorders, precursors to atherosclerosis [69]. Recently, Xiangshan Xu et al. (2020), showed in the pre-clinical protocol in human umbilical vein endothelial cells, HUVEC’s that even with low shear vascular cell stress it was possible to activate the pro-inflammatory pathway which may occur through the signal transducer and activator of transcription 3 (STAT3), suggesting some DAMPs are mediated by miR-181b-5p [70].

Endothelial cell activation is the initial stage characterised by intravascular endothelial disturbance, which is followed by pre-inflammatory changes. The vascular endothelium is a potentially more prone acceptor of endogenous substances that work as DAMPs inducing vascular pro-inflammation since its extensive surface area (~3000 to 6000 m^2^) makes this thin luminal surface very vulnerable to adverse developments [71]. Also known as sterile inflammation, pro-inflammatory insult corresponds to the immune-inflammatory response characterised by the production and maturation process of pro-inflammatory mediators such as interleukins 1β and 18 (IL1 β and IL18), as well vascular endothelial cells (ECs) plasma membrane pore formation through protein gasdermin-D (GsdmD) cleavage into N-terminal fragments, known as pyroptosis [72,73,74].

### 3.2. Inflammasome Activation

The intracellular multiprotein platform (inflammasome) is the best-characterised player responsible for carrying out proteolytic cleavage activation in an inactive zymogen cysteine-aspartic protease (pro-caspase-1) also known as interleukin-1 converting enzyme. Due to its action on the axis, IL1β/IL18/GsdmD, the inflammasome is considered a primary pyroptosis regulator. PRRs such as inflammasome-forming, the Nod-Like Receptor Protein 3 (NLRP3) coupled with apoptosis-associated speck-like protein containing a caspase activation and recruitment domains (ASC) protein and pro-caspase-1 inflammasome form multiprotein intracellular sensor stress [75]. As part of this process, the interleukin transcription is upregulated through Toll-Like receptors (TLR Family) associated with the myeloid differentiation primary response 88 (MyD88) protein with signal transmission to the IKK kinase complex to heterodimer nuclear factor kappa-light-chain-enhancer of activated B cells (NF-κB) cell nucleus translocation. Therefore, when not stimulated, the NF-kB factor found in the cytoplasm binds to an inhibitory complex IkB; thus, the phosphorylation process is needed for translocation to happen. However, this does not exclude the possibility of other forms of interleukin synthesis via proteolytic action in the cytosol by calcium-dependent cysteine proteases (calpain) or via inflammatory caspases-11/4 during vascular disorders [76,77,78].

Most studies investigating microRNAs in overall cardiovascular remodelling are, in fact, limited if not deficient in their in vivo findings; however, the complexity of the underlying mechanisms, coupled with the increasing importance of the research on this topic, has significantly driven, and subsequently improved, our understanding of microRNA actions in vascular remodelling as a whole [79].

### 3.3. miRNA in Pyroptosis Mechanism

miRNAs are essential post-transcriptional-mediated gene expression regulators in VECs [79]. Usually, miRNAs have their functions appraised through pre-clinical models verifying protein expression changes associated with specific genes [80]. In many of these scientific protocols, the dubiousness of circulating or intracellular sequences of miRNAs shows both anti-inflammatory and pro-inflammatory manifestations. Moreover, if pro-inflammation, per se, is linked by micro-endogenous species (alarmins) DAMPs-like, then microRNAs with a nano-size should be more relevant biomolecules able to carry changes in the cellular metabolism, even because DAMPs itself could also be a miRNA’s source.

In the last years, studies have shown the pro-inflammatory pathway has been under the influence of some miRNAs acting as modulators of the NLRP3 inflammasome expression or other pyroptotic molecules related [81]. The miR-223 upregulated in melatonin-treated human aortic endothelial cells (HAECs) reduced the NLRP3 inflammasome expression after stress induced by ox-LDL working as a DAMP-like exposition [82]. In HAECs, the ox-LDL stress inducer exposed over 24 h showed miRNA-30c-5p playing an inhibitory effect on NLRP3 inflammasome expression through the forkhead box O3 (FOXO3 or FOXO3a) pathway [83]. In a high glucose concentration medium. In an experimental hyperglycaemic protocol, using a high-glucose medium and DAMP-like sugar crystals as stressors, miR-22 was observed to negatively regulate NLRP3 inflammasome expression in human endothelial cells (EA.hy 926). The same study found the inverse expression of the lncRNA MALAT1 [84]. Although all miRNAs discussed above showed negative regulation in the pyroptosis pathway, the study conducted in HUVECs alsoexposede to ox-LDL showed increases in the expression of miR-125a-5p related to increased pro-inflammatory effects via tet methylcytosine dioxygenase 2 (TET2) pathway, thus showing opposite effect [85].

According to recent data concerning myocardium integrity outcomes, miRNAs also play a relevant dichotomy in functionally mediating pro-inflammation and anti-inflammation effects impacting cell survival. The murine diabetes-induced model by intraperitoneal injection of streptozotocin (STZ) and the primary cardiomyocytes in vitro-based model exposed to high glucose levels increased the miRNA-30d expression mediating FOXO3a pathway suppression [86,87]. Likewise, carried into two in vitro pre-clinical models the protocol with elevated glucose levels in mouse macrophages RAW 264.7 and human ventricular cardiomyocytes (diabetic vs. non-diabetic) showed the microRNA-9 upregulated less damage associated with the NLRP3 and caspase-1 lower expression mediatedV Like RNA Binding Protein 1 (ELAVL1) signalling [88,89]. In experiments conducted in a vitro model with the primary culture of neonatal mice ventricular cardiomyocytes through stress induced with hydrogen peroxide (H_2_O_2_) and in vivo mutant model of overexpressing miR-135b in myocardial infarction (MI) protocol during 24 h of artery occlusion, the authors found that the upregulation of miR-135b provided cellular protection in both protocols [90], found through methods such as molecular expression, morphological changes, and contractile function evaluation the relationship between the downregulation of the pyroptosis pathway through axis NLRP3/caspase-1 and miR-703 upregulation expression. The increased miR-703 also showed protection in the cell culture of mouse cardiomyocytes from pyroptosis damage related to the hypoxia followed by reoxygenation (H/R) protocol [91].

In contrast, higher levels of miR-155 carried by exosomes from macrophages RAW264.7 showed important mediation in pyroptosis damage associated with hypertrophy development in hearts with uremic manifestation [92]. The vivo model submitted to the myocardial ischemia-reperfusion (I/R) protocol showed that miR-149 is related to the deleterious effect of the pyroptosis pathway through the downregulation of the FOXO3 pathway [93]. Lastly, in two pre-clinical models of the embryonic cardiomyocyte cell line (H9C2) submitted to the H/R protocol and rodent hearts under the myocardial I/R protocol, miR-132 showed elevated expression associated with oxidative stress. Also, the stress induction related to pyroptosis effects was mediated by the NAD-dependent deacetylase sirtuin-1 (SIRT1) protein enzyme responsible for multiple functions in cell metabolism control [94].

In a recent relevant study related to pyroptosis gene biomarkers, the miRNA-mediated pyroptosis pathway in humans comes with a clinically randomised, double-blind placebo-controlled trial published by Neda Roshanravana et al. (2020) in a type 2 diabetes population. The volunteers received oral administration with sodium butyrate and inulin over 45 days to intend to provide pro-inflammatory marker reduction. Among the multiple data, it was found the negative correlation with miRNA-146a-5p and miR-9-5p both associated with axis NLRP3/caspase-1/IL1β expression reduction which may establish an important tool for future studies concerning biomarkers that can cross-talking diseases and the pyroptosis pathway [95,96].

## 4. miRNAs and Vascular Endothelial Dysfunction

Vascular endothelium consists of a single-layer inner of ECs in a juxtaposed arrangement, taking place in the organism as a systemic interface between the bloodstream and tissue organs. Vascular endothelium cells (VECs) have essential biological functions, interacting actively with blood components in the fluid phase through membrane receptors and selective permeability functions. This capacity allows vessel structural changes through vascular tone control (e.g., dilatation/constriction) and vascular smooth muscle cell (VSMC) growth and proliferation. This process also triggers extracellular matrix synthesis, platelet recruitment, and foam-cell formation, which together alter the local mesenchymal environment and amplify macrophage infiltration during inflammation [97,98,99]. Notably, many of these endothelial functions are regulated by microRNAs, which help maintain vascular homeostasis and promote recovery after injury. For example, endothelial injury induces TGF-β activation, which in turn stimulates the MAPK/ERK signalling cascade (mitogen-activated protein kinase/extracellular signal-regulated kinase), driving endothelial proliferation and repair [100].

The multifactorial nature of endothelial dysfunction underpins a spectrum of interrelated cellular and molecular events. This proliferation of studies reflects the field’s rapid expansion but has paradoxically left the core pathobiology of endothelial impairment incompletely defined with significant gaps in our understanding and remains not entirely elucidated to date. These efforts include the unravelling of the pathophysiological mechanisms alongside the biomolecular responses underlying endothelial dysfunction and its relationships with inflammatory processes, lipids, and vascular smooth muscle cells (VSMCs) in according to Romaine et al. (2015), understanding this complex interplay is of great importance [101].

For this purpose, we are going to bring recent studies using miRNAs as an important target for endothelial dysfunction treatment.

### 4.1. The Vascular Endothelial Cell in Activation State

As expected, there is a complex myriad of mechanisms related to phenotypic maintenance of vascular endothelium homeostasis. The set of alterations due to ongoing pro-inflammatory and pro-coagulant processes resulting in significant changes in baseline parameters are characterised as cell activation endothelial states, a term coined in the 1960s related to identifying loss of vascular integrity [100]. Moreover, this condition is evidenced by several authors as the prior stage of endothelial dysfunction impairment [101].

### 4.2. Vascular Endothelial Dysfunction Evaluation

The prevalence of macro- and microvascular endothelial dysfunction is elevated in the population who have been diagnosed with cardiovascular diseases, showing a tendency for pathological effects progression according to the number of risk factors [102,103]. In this context, the clinical investigation mainly for scientific purposes has been approaching two types of protocols: a non-invasive one by verification of the dilating vessel capacity during reactive hyperaemia followed by ischaemic occlusion in upper and lower limbs, and the invasive one through blood collection samples to evaluate circulating biomarkers (e.g., nitrates and nitrites). In fact, it has been very often that both protocols have been applied concomitantly in order to characterise the endothelial dysfunctional state [104,105].

### 4.3. Nitric Oxide Biosynthesis in Vascular Endothelial Cell

Chronic diseases such as diabetes, dyslipidaemia, hypertension, obesity, heart failure, atherosclerosis or poor habits, such as smoking, sedentarism or the ageing process, are all related to vasodilatation disorders [106]. In favour of those vasoreactivity effects, the production and bioavailability of the soluble gas nitric oxide (NO) obtained from the L-arginine aminoacidic substrate plus oxygen and cofactors [e.g., reduced nicotinamide adenine dinucleotide phosphate (NADPH), tetrahydrobiopterin (BH4), flavin mononucleotide (FMN), flavin adenine dinucleotide/mononucleotide (FAD), and iron protoporphyrin IX (heme)] are synthesised in L-citrulline and NO by the endothelial nitric oxide synthase (eNOS) isoform playing a central role in the pathogenesis of endothelial dysfunction. eNOS activity might be controlled by the calcium-dependent mechanism through the activation of the enzyme calmodulin (CaM) or independently via protein kinase A (PKA) and protein kinase B (AKT) pathways [107]. In this context, it was found the AKT/eNOS axis was negatively modulated by miR-615-5p through the gene’s expression encoding insulin-like growth factor 2 (IGF2) protein and encoding the ras association domain protein family member 2 (RASSF2) protein showing cell injury and 60% NO expression reduction in HUVECs. The authors also showed in vitro VECs that the growth expression and angiogenesis process were also impacted by miR-615-5p elevation [108]. However, a study published by Jing-Bo Li et al. (2017) showed a possible dubious play role in the Akt-activation, due to a contradictory deleterious process related to increasing miR-138 expression in HCAECs caused by ox-LDL injury exposition through axis PI3K/AKT/eNOS upregulation. The authors suggested that excess NO production may lead to lipid peroxidation in this model, thus, increasing the expression of pro-inflammatory cytokines and cellular injury [109].

According to the high diffusion from the VECs to intracellular space in vascular smooth muscle cells (VSMCs), the NO free radical molecule might bind to cytosolic guanylate cyclase also known as soluble guanylate cyclase (sGC) inducing catalytic action for conversion guanosine-5-triphosphate (GTP) to cyclic guanosine-3′,5′-monophosphate (cGMP). Moreover, the signal to GTP conversion may also directly target the transmembrane guanylate cyclase receptors. The cGMP is an essential metabolite in VSM tissue relaxation acting as a secondary messenger mediating transcription change factors such as cAMP-response element-binding protein (CREB), serum response factor (SRF), and nuclear factor of activated T-cells (NFAT) or post-transcriptional changes through cGMP-dependent protein kinase G (PKG) activity. Lastly, PKG triggers downstream signalling through cyclic nucleotide-gated cation channels, cGMP-regulated hydrolysing phosphodiesterase, and vasodilator-stimulated phosphoprotein pathways, all crucial mechanisms in the cardiovascular system mainly due to the impact associated with vascular dilatation [110,111].

### 4.4. miRNAs Interacting with Endothelial Nitric Oxide Synthase

The lack of eNOS expression is one of the main reasons identified in endothelial dysfunction. Similarly, miR-195 and miR-582 bonded to the 3′ untranslated region of the gene nitric oxide synthase 3 (NOS3) located on chromosome 7q36 reduced eNOS mRNA transcription reverberating in less NO production in the culture of microvascular endothelial cells (MVEC) [112]. Likewise, the miR-24 reduced the eNOS as well as specific protein 1 (Sp1) expression in HUVECs compromising NO biosynthesis and the cellular proliferation pathways [113]. Besides, acute and chronic detriment NO availability caused by pathological manifestation or trauma may suffer the second impact due to oxidative stress. The accumulation of reactive oxygen species (ROS) through anion superoxide converting in peroxynitrite (ONOO−), an oxidative molecule to the BH4 cofactor might induce eNOS decoupling [114]. In a pre-clinal study, using statin pharmacological treatment, it was found that increased expression of miR-133a in VSMCs corresponded with decreased GTP cycle hydrolase 1 (GCH1) enzyme expression which is associated with BH4 biosynthesis. Peng Li et al. (2016) carried out several analyses in vitro and vivo models suggesting that the miR-133a may be a relevant trigger for statin in order to prevent eNOS decoupling, paving the way for possible endothelial dysfunction therapy through oxidative stress modulation [115].

The dysregulation process in oxygen transportation and ROS production after IR injuries may impact microcirculatory function related to communication between cardiac microvascular endothelial cells (CMVECs) and cardiomyocytes. In in vitro models of cardiomyocytes under the hypoxic-preconditioned protocol, it was observed an increased expression of miRNA circHIPK3 was observed by exosomes. In addition, the circHIPK3 transportation revealed downregulating of miR-29 expression associated with ROS reduction and apoptosis program-cell-death reduction in parallel with increased Insulin-like growth factor 1 (IGF-1) expression [116]. Intracellularly, ROS might play as a DAMP entity starting transcription and activation of cytokine via the pyroptosis pathway. Therefore, the pro-inflammatory signal also leads to endothelial cell activation in VECs also might contribute to vascular endothelial dysfunction [101].

### 4.5. Vascular Endothelial Dysfunction Biomarkers

In an attempt to identify possible biomarkers for endothelial dysfunction, Khalyfa et al. (2016) analysed 84 miRNAs into two groups of volunteer obese children, with normal vascular endothelial functions versus vascular endothelial dysfunction. Next, three microRNAs were found related to vascular endothelial dysfunction; the miR-125-5p and miR-342-3p were closely related to hypertensive conditions, and miR-365-3p was also related to myocardial remodelling due to abnormal functionality of left ventricular evidenced. According to bioinformatics analysis, the miRNAs were correlated with the cardiac metabolic control pathway due to the influence of peroxisome proliferator-activated receptor γ (PPARγ) and activin A type IIB receptor (ACVR2B) mediating the transforming growth factor-beta (TGF-β) signalling as well as Angiotensin II-Forming (Ag II), tumour necrosis factor-alpha (TNF-α), and IL-1β inflammatory pathway [117]. PPAR-γ plays an important mediator in atherosclerotic damage relief through the increase in miR-27 expression. In human artery endothelial cell (HAEC) culture exposure to ox-LDL and antioxidant urolithin A (UA) showed expression reduction of inflammatory cytokines and an increase in e-NOS expression through downregulating extracellular signal-regulated kinase (ERK) [118].

Circulating miR-181b and miR-223 levels predict early endothelial dysfunction in hypertensive patients, warranting prospective validation in clinical cohorts. Moreover, antagomir-mediated inhibition of miR-33 in preclinical models restores ABCA1-dependent cholesterol efflux, underscoring miR-33 antagonists as viable therapeutic candidates.

### 4.6. Vascular Endothelial Dysfunction by Pathomorphological Manifestations

**Figure 1:**
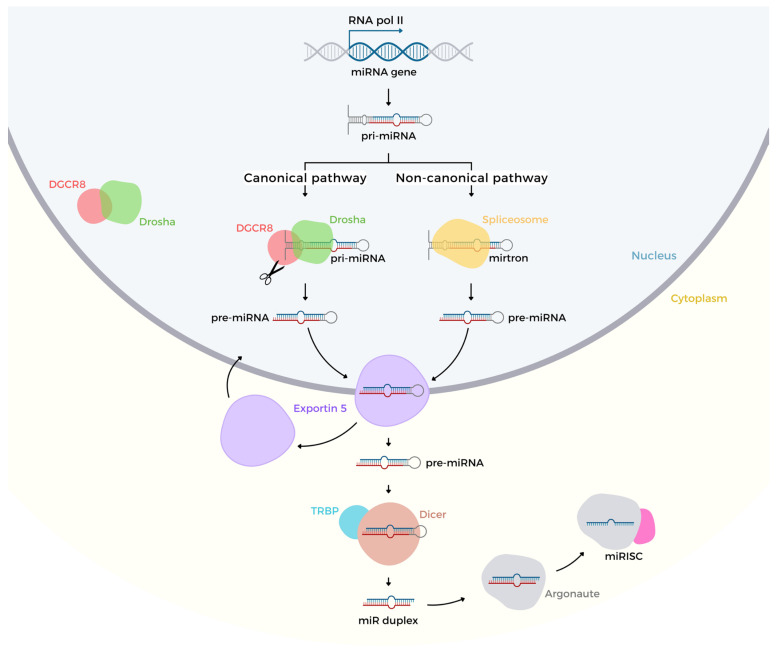
Implications of MicroRNA Biogenesis in Endothelial Dysfunction and Vascular Disorders. Illustration of the biogenesis pathways of microRNAs (miRNAs) and their relevance to cellular regulation, with a focus on endothelial nitric oxide synthase (eNOS) modulation and vascular health. MiRNAs are small, non-coding RNAs that regulate gene expression post-transcriptionally. The canonical pathway begins with the transcription of miRNA genes by RNA polymerase II, producing primary miRNAs (pri-miRNAs). These are processed by the Drosha-DGCR8 complex into precursor miRNAs (pre-miRNAs), which are exported to the cytoplasm via Exportin-5. In the cytoplasm, Dicer and TRBP further process pre-miRNAs into miRNA duplexes. One strand of the duplex is incorporated into the miRNA-induced silencing complex (miRISC), guided by Argonaute proteins, to regulate target mRNA. In the non-canonical pathway, some pre-miRNAs bypass Drosha processing and are derived from splicing events, such as mitrons. These alternate pathways highlight the diversity of miRNA processing and its potential dysregulation in pathological states. Specific miRNAs, such as miR-615-5p and miR-138, are further discussed in the review for their roles in modulating the AKT/eNOS pathway, influencing nitric oxide (NO) production, and contributing to endothelial dysfunction. Understanding these pathways provides insights into the mechanisms underlying chronic diseases like diabetes, hypertension, and atherosclerosis, and highlights the therapeutic potential of targeting miRNAs in vascular disorders (Created by BioRender.com).

The hyperplasia and neointima formation are part of a pathophysiological process leading to endothelial dysfunction. Moreover, it has been while studies suggesting the low bioavailability of NO, and eNOS lack of expression are related to this process as reviewed by Ahanchi et al. (2007) [119,120]. Vascular endothelial dysfunction might be induced by physical actions in the intimate wall of the endothelium, such as blood shear, which could induce inflammatory and fibrotic processes both associated with TGF-β signalling. In inflammatory settings, upregulation of the TGF-β1 signalling cascade promotes endothelium-mesenchymal transition (EndMT) and neointimal hyperplasia via downregulation of the protective kinase MAPK7 through miR-374a. This shift impairs endothelial function and vascular compliance, predisposing to adverse clinical outcomes [121]. Moreover, TGF-β1 induces miR-143 and miR-145, which inhibit hexokinase II (HKII) and integrin β8 (ITGβ8), thereby modulating angiogenesis and vessel stabilisation. Finally, in an in vitro co-culture system, smooth muscle cell (SMC)–to–endothelial cell (EC) cross-talk amplifies these miRNA-mediated inflammatory signals, highlighting how intimal inflammation drives phenotypic alterations that disrupt regional vascular dynamics [122]. Physical stress caused by vascular endothelial stretching (which is susceptible to ruptures), along with increased shear stress, leads to an adverse scenario characterised by the loss of intimal integrity and is associated with severe cardiovascular disease (CVD) events. In this sense, it was carried study using mechano-transduction pathway-induced protocol through the polyacrylamide hydrogels usage in HUVECs culture. The results showed the transient receptor potential cation channel subfamily V member (TRPV4) mediating calcium influx through miR-6740 as well as endothelin-1 (ET-1) upregulation [123] (Figure 1).

## 5. miRNAs and Atherosclerosis

Atherosclerosis is a progressive and chronic disease which may develop systemically vascular lesions resulting in coronary syndrome, strokes and contribution to vessel aneurysm formation [124]. The pathogenesis mechanism of atherosclerosis is characterised by the thickening of the tunica intima through the accumulation of lipoprotein molecules mostly ox-LDL, invading the vascular lumen as it reduces the space of blood passage due to narrowing in the vessel light, consequently, causing lesions and inflammatory processes [125]. According to the innate immune system signalling, monocytes are recruited to the injured area and adhere to endothelial cells in an active state, invaginating the subendothelial vascular space. Nevertheless, the monocytes differentiate the process into macrophages, increasing lipids from lipoproteins uptake increased by this way altering to cells foamy form, which, when accumulating, can form streaky fatty followed by atherosclerotic lesions (atheroma). During the atherosclerosis disease’s progression, a consistent inflammatory downstream allows more ox-LDL retention also inducing VEC’s extracellular matrix changes until its rupture, consequently thrombi formation and vessel blockage.

### 5.1. Pre-Clinical miRNA Study in Atherosclerosis Protocols

Notably, translational research has limitations concerning the complexity associated with the role of miRNA’s regulation interacting with chronic and systemic diseases such as atherosclerosis. To elucidate this challenging scenario in vivo only in mice models widely used for scientific purposes, Moritz von Scheidt et al. (2017) found 263 atherosclerosis pathways in the literature with multiple candidate genes sharing some level of disease relationship [126]. In any pathway described extremely high amounts of miRNAs could play different regulations depending on the disease stage context. However, the Gene Ontology Consortium and miRBase are efforts to catalogue the majority of small non-coding sequences, unveiling features and in order to identify and validate new therapeutic targets [127,128]. Currently, there are clinical trials carrying protocols of treatment based on miRNA’s function [129], although we have not already made progress in the CVD field even during the advanced stage of atherosclerosis. In this sense, we will show potential recent findings that correspond to advances in miRNA research applied to atherosclerosis diseases.

Identifying the relationship between post-transcriptional regulation and phenotypic changes is the primary importance during the screening for new miRNAs. For this purpose, pre-clinical (in vivo/in vitro) is easily reproducible although samples coming directly from human-affected tissues (in vivo/post-mortem) are fortuity genetic material sources. According to that, it was found in tissue vessels with atherosclerotic plaques; there is miR-181a increased expression from 3 to 4 times compared to healthy tissue vessels. The same study showed anti-apoptotic B-cell lymphoma 2 (Bcl-2) protein family had decreased expression while high miR-181a maintained high levels evidenced in HUVEC’s culture, suggesting a new anti-apoptotic pathway therapy mediated by this miRNA sequence [130]. On the other hand, in the context of human diseases being reproduced in animals, the pre-clinical rabbit in vivo model was the first applied in atherosclerosis studies. Particularities in the lipid metabolism of rabbits, such as the LDL and VLDL receptors expression behaviour in hypercholesterolemia, make these mammals closer to humans when compared to rats or mice in atherosclerosis [131,132]. To abbreviate the period in atheroma formation in an atherosclerosis study in rabbits, the researchers Feng Zhang et al. (2018) applied the technique of endothelial injury with a catheter coupled to a latex balloon, this physical method was conducted in combination with a high-fat diet protocol. Surprisingly, dozens of miRNAs with altered expression were found, although at least seven circulating miRNAs were catalogued related to atherosclerotic plaque formation [133].

### 5.2. miRNA Targeting Lipid Metabolism

Hypercholesterolemia is a risk factor well-established in atherosclerosis; therefore, pre-clinical protocols which could reproduce a saturated lipid environment may assist studies at the cellular level. The ox-LDL has been consistently used to induce oxidative stress through a rich lipids/phospholipid mean carried on several cell lineages. In Michel Desjarlais et al. (2017) study was carried hypercholesteraemic protocol in HUVEC’s culture exposed to ox-LDL, and apolipoprotein-E (APO-E) knock-out mice showed the miR-150 expression reduction. The authors also demonstrated by several methods with anti-miR treatment, silencing RNA technique, and miR-150 agonist, when the availability of miR-150 increased the proto-oncogene tyrosine-protein kinase (SRC) kinase signalling inhibitor-1 pathway is downregulated. Thus, SRC protein activity could indirectly target the miR-150 after EC injury [134]. Following a similar line, the experiments carried out on both HUVECs and HCAECs cultures exposed to ox-LDL showed an increase in miR-758 expression. It was also found the expression reduction in succinate receptor 1 (SUCNR1), responsible for ATP biosynthesis, and vascular endothelial growth factor (VEGF) is associated with angiogenesis and SATA3 phosphorylation.

The Wang et al., study confirmed the upregulation of miR-758 in the atherosclerotic model of vascular ECs from APO-E knock-out mice fed with a high-fat diet. In an alternative cell lineage study, human monocytic (THP-1) culture was submitted under ox-LDL exposition and showed the increased expression of the lncRNA-NEAT1 proportionally with inflammatory cytokines elevation. The same protocol showed the upregulation of miR-342-3p provides a counterbalance through pro-inflammatory cytokines IL-6, IL-1β, TNF-α and COX-2 reductions. Also, by blocking both non-coding sequences, the authors showed a significant reduction in lipid uptake, suggesting that both miR-342-3p and lncRNA-NEAT1 mediation are related to lipid metabolism control [135].

**Figure 2:**
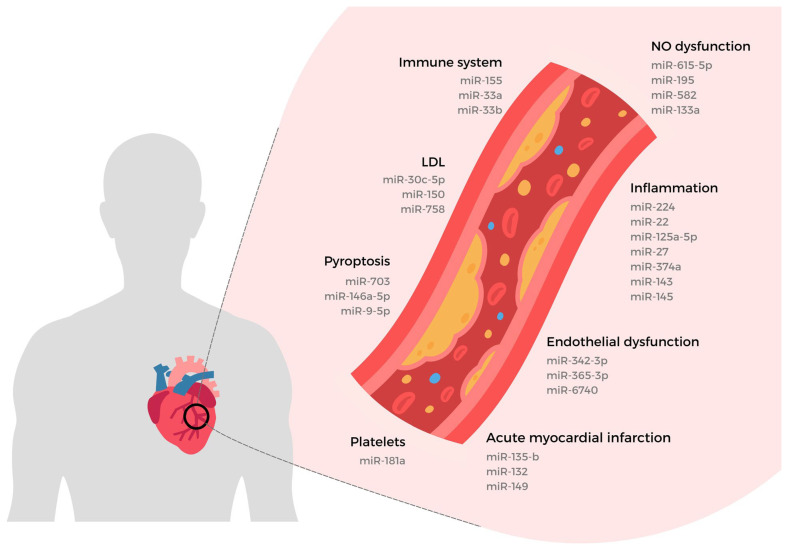
MicroRNA Signatures in Cardiovascular Pathophysiology: Linking Endothelial Dysfunction, Inflammation, and Acute Myocardial Infarction. Highlights the diverse roles of specific microRNAs (miRNAs) in cardiovascular pathophysiology, providing a schematic overview of miRNAs implicated in various processes, including nitric oxide (NO) dysfunction, inflammation, immune response, endothelial dysfunction, pyropoptosis, platelet activity, and acute myocardial infarction. Each category is associated with distinct miRNA signatures: NO Dysfunction: miR-615-5p, miR-195, miR-582, and miR-133a regulate NO bioavailability, influencing vasodilation and endothelial health. Inflammation: miR-224, miR-22, miR-125a-5p, and others contribute to pro-inflammatory signalling and vascular damage. Immune System: miR-155, miR-33a, and miR-33b modulate immune responses critical to atherogenesis. Endothelial Dysfunction: miR-342-3p, miR-365-3p, and miR-6740 impair endothelial function, leading to vascular complications. Pyroptosis: miR-703, miR-146a-5p, and miR-9-5p are involved in programmed inflammatory cell death, exacerbating tissue injury. Platelets: miR-181a plays a role in platelet activation and aggregation. Acute Myocardial Infarction: miR-135-b, miR-132, and miR-149 are associated with ischemic injury and cardiac remodelling. The aforeillustrated is a visualisation of the synthesises and the complex interplay of miRNAs in cardiovascular health, demonstrating miRNAs as critical biomarkers and therapeutic targets (Created by BioRender.com).

Cholesterol metabolism critically influences atherogenesis through reverse transport and efflux mechanisms that preserve endothelial integrity by regulating free cholesterol in peripheral tissues and subendothelial macrophages. The miR-33a/b isoforms have been intensively studied for their capacity to elevate circulating HDL, the primary cholesterol acceptor, an effect frequently associated with reduced atherosclerotic burden. Notably, a clinical trial involving 30 participants with and without coronary artery disease (CAD) revealed higher miR-33 levels in non-CAD subjects, supporting its potential as a circulating biomarker [135]. However, contradictory evidence emerges from translational studies: Näär et al. (2018) highlighted that miR-33 blockade in LDL-receptor knockout mice and Price et al. (2019) induced adverse metabolic shifts, including moderate hepatic steatosis and elevated serum triglycerides during long-term high-fat feeding [136,137]. This context-dependent duality, protective HDL elevation versus detrimental metabolic side effects, underscores the translational challenges of therapeutic miR-33 modulation (Figure 2) [138,139].

## 6. Conclusions

This review aimed at the relevant recent findings concerning the interaction of miRNAs with the pathophysiological mechanism in pro-inflammation, endothelial dysfunction and atherosclerosis. All studies presented contribute to molecular pathways cross-talking understanding through evidence-based research related to miRNAs as therapeutic targets or disease biomarkers. We believe this biomedical resource will be widely explored as functional understanding advances across various fields of study. Elucidating the specific mechanisms through which miRNAs regulate key biomolecular interactions and processes in atherosclerosis remains a critical objective for future research.

## Figures and Tables

**Table 1:** Biological function from ncRNAs.

ncRNA; Nucleotides Numbers; Classification	Putative Functions	Reference
miRNA (18–23, small nc RNA)	Translational repression; Transcriptional activation	Nielsen & Holmstrøm (2013) [21] Vemuganti et al. (2014) [22]
tiRNA (14–30, small nc RNA)	Transcriptional initiation	Vemuganti et al. (2014) [22]
siRNA (19–25, small nc RNA)	mRNA degradation	Meister & Tuschl (2004) [23]
tasiRNA (20–24, small nc RNA)	Gene silencing in plants	De Felippes (2017) [24]
tel-sRNA (23–28, small nc RNA)	Epigenetic regulation of telomerase	Frenk et al. (2019) [25]
rasiRNA (24, small nc RNA)	DNA-methylation	Silva et al. (2017) [26]
piRNA (24–32, small nc RNA)	Transposon mobilisation	Ding et al. (2019) [27]
CRISPR (24–48, small nc RNA)	Prokaryotic immune control	Huescas et al. (2019) [28]
TSS-miRNAs (20–90, medium-size ncRNA)	Transcriptional regulation	Liu et al. (2017) [29]
PASR (20–200, medium-size ncRNA)	chromatin modifications	Ma et al. (2017) [30]
snoRNA (60–300, medium-size ncRNA)	Maturation of other ncRNAs	Abel & Rederstorff (2019) [31]
scaRNA (83–330, medium-size ncRNA)	Guiding spliceosomal RNAs	Ratner et al. (2019) [32]
Long ncRNAs: lncRNA (>200, long ncRNAs)	Transcriptional regulation	Silva et al. (2017) [26]
T-UCR (200–779, long ncRNAs)	Antisense inhibition of mRNAs and ncRNAs	Sun et al. (2020) [33]
CUT (200–800, long ncRNAs)	Chromatin regulation	Vera & Dowell (2016) [34]
SUT (200–800, long ncRNAs)	Transposon silencing	Xu et al. (2009) [35]
TERRA (100–9000, long ncRNAs)	Regulation of telomere length	Marión et al. (2019) [36]
PROMPT (500–2500, long ncRNAs)	Promoter control	Preker et al. (2011) [37]
